# An evaluation of 3D modelling in patients with locally advanced pelvic malignancy undergoing surgical resection

**DOI:** 10.3389/fonc.2025.1719458

**Published:** 2026-01-12

**Authors:** Rachael Elizabeth Clifford, Victoria Fretwell, Sadia Gul, Amanda Coop, Phillip Borg, Hamish Clouston, Paul A. Sutton

**Affiliations:** 1 The Christie NHS Foundation Trust, Manchester, United Kingdom; 2 Division of Cancer Sciences, University of Manchester, Manchester, United Kingdom

**Keywords:** rectal, cancer, pelvic exenteration, consent, anal

## Abstract

**Background:**

Surgery for locally advanced pelvic malignancy is complex, requiring careful operative planning and effective patient consent. Traditional two-dimensional (2D) imaging can limit understanding of tumour-organ relationships for both clinicians and patients. Three-dimensional (3D) reconstructed imaging may have a role in enhancing surgical planning, patient understanding and trainee education.

**Methods:**

Adult patients with locally advanced rectal or anal cancer undergoing planned resection at a UK tertiary cancer centre were prospectively identified. CT scans were reconstructed into interactive 3D models using the Visible Patient™ platform. Models were reviewed by surgeons and trainees prior to outpatient consultations and then demonstrated to patients during the consent process. Surgeons, trainees, and patients completed structured questionnaires incorporating Likert-scale responses and free-text feedback. Qualitative data were analysed thematically.

**Results:**

Fifteen patient-specific pelvic models were created, with feedback obtained from all surgeons and trainees and from 13 patients. Patient responses were overwhelmingly positive, with median scores of 9–10/10 for realism, understanding of disease and surgery, and perceived benefit during consultation. Thematic analysis highlighted improved understanding, empowerment, and engagement in decision making. Surgeons rated the models moderately for operative planning but highly for trainee education and patient communication. No operative plans were altered following model review. Trainees reported strong educational value, while radiologists noted limitations in segmentation accuracy for intraluminal disease.

**Conclusion:**

CT-based 3D reconstructed pelvic models provide substantial benefit in patient education and shared decision making for complex pelvic cancer surgery, with additional value as a training tool. Further studies are warranted to define their role in operative planning and cost-effective implementation.

## Introduction

Pelvic surgery in patients with locally advanced rectal cancer provides the surgeon with multiple challenges in terms of operative approach, the need for multi-visceral resection, and ascertaining the relevant patho-anatomy. Understanding the interaction of the tumour with the pelvis beyond the mesorectal fascia is an essential requirement for operative planning and execution, given the multiple viscera and neurovascular structures present within a rigid confined space (1). Shared decision making with patients to obtain informed consent is a critical part of preparing them for surgery (2). Decision aids, such as reviewing scan images, diagrams or written information are often utilised to complement a patient’s active involvement in decision making whilst respecting personal values (3). It is estimated that 40-80% of information verbally discussed with a patient during a consultation about surgical planning is often forgotten due to the additional stress (4, 5). Novel approaches are needed therefore to effectively convey the complexity of surgery to patients as well as helping them understand the associated risks.

For accurate cancer diagnosis and staging, CT and MRI scans are utilised. Although these techniques have progressed significantly over the last decade, images are presented in 2 dimensions (2D), meaning that a thorough appreciation of the relations of a tumour with its surrounding viscera, often in planes already exposed to chemoradiotherapy, can be challenging. Surgical planning requires the ability to conceptualise the surgical field and planes in 3 dimensions (3D). These challenges extend into the outpatient clinic, where explaining the nuances of locally advanced disease to patients with only 2D visual aids can be extremely challenging. The development of technologies to re-format CT scans into 3D has the potential to revolutionise surgical planning and patient consent, as well as support the education and development of surgical trainees. 3D reconstructive models have been shown to be especially useful in challenging areas, such as the pelvic side wall, by ensuring appropriate planning to maximise the likelihood of clear margins whilst avoiding unnecessary morbidity (6). We aimed to assess the role of 3D reconstructed CT scan images in surgical planning, the patient consent process, and trainee education in patients undergoing surgery for complex pelvic malignancy.

## Methods

Patients were identified through the advanced pelvic cancer MDT at The Christie NHS Foundation Trust, a tertiary cancer hospital in the UK. Inclusion criteria included patients over the age of 18 years with a diagnosis of locally advanced rectal or anal cancer, who were suitable for resection and able to provide informed consent. Patients with primary and recurrent disease identified between September 2023 and June 2024 were eligible, with a pragmatic approach to patient selection based upon having sufficient time available to create and fully evaluate the model prior to surgery.

CT scan images were reconstructed using the Visible Patient™ platform (7). This software enables structures to be colour coded and added and removed as required. The 3D images were reviewed by the surgeon and colorectal surgical fellow individually before being demonstrated to the patient in the outpatient clinic setting, with an evaluation recorded by each group through a dedicated study questionnaire. Participants were asked to grade questions using a score of 1–10 or Likert scale analysed by summary statistics, or free text boxes subjected to inductive thematic analysis. The study was registered as a quality improvement project at The Christie Hospital and funded by The Christie Charity, in partnership with NIHR Manchester Biomedical Research Centre.

## Results

The pelvis of fifteen patients was modelled, with evaluations returned from all surgeons and trainees, as well as 13 patients. Eleven patients were male with a median (range) age of 63 years (33–76) years. All patients were planned for beyond TME surgery due to locally advanced rectal or anal cancer: six soft tissue pelvic exenteration, four high complexity total pelvic exenteration (including resection of neurovascular structures and/or bone), two extended abdomino-perineal resections, and one rectal resection with *en bloc* total abdominal hysterectomy.

Patient feedback was overwhelmingly positive, with all patients offering a sore of 9 or 10/10 when asked if the model appeared realistic, if it had been helpful in enabling them to understand their condition, and whether they benefited from the use of the model during their consultation. Only two patients reported that the models initially made them feel uncomfortable to see the position of their tumour. A thematic analysis of the free text comments identified: empowerment through understanding (e.g. of the anatomy and why each additional structure had to be excised), increased control (e.g. feeling more empowered within the decision making process), and positivity (e.g. specifically commenting on the bright contrasting colours). A summary of some key comments and the median scores are summarised in **Table 1**.

**Table 1:** Median scores and example comments from patient participants.

Question	Median score (out of 10) (range)	Example participant comments
This would be useful to my surgeon in planning my operation	9.8 (8-10)	“Adds additional explanation and precision to what is done” [P4] “Definitely, I’d want to know why they haven’t referred to it if it’s already available” [P10]
The model looks realistic	9.8 (9-10)	“It looks realistic and confirms what will be removed” [P7] “Yes and even with little anatomy knowledge I could figure it out with some guidance” [P9]
I like the models	9.9 (9-10)	“Allows me to visualise what needs to be done and why” [P10] “Helped me to understand the proximity of the structures, the procedure planned and its necessity” [P11]
The models will help me understand my condition	9.9 (9-10)	“Yes, definitely, when you see it on the model you see where it is, where it is lying and what it is pressing on” [P2] “Yes, made a big difference, you can see how close the tumour is to the other structures” [P12]
The models will help me understand my operation	9.8 (9-10)	“Yes, makes me understand why all the organs and sacrum needs to be removed to get clear area and hopefully live longer” [P12] “The model made me realise the requirement to remove the structures like the bladder and prostate” [P13]
The model makes me feel squeamish or uncomfortable	2.5 (1-10)	“No this doesn’t make me, it might for some people, but gives a better idea of what’s happening and how difficult it is to get at it” [P4] “I wondered at first what it would be like, the grey shades on the traditional CT make me feel worse” [P12]

Patients were also asked how informed they felt about treatment options using a traditional decisional conflict scoring system (The Ottawa Hospital (8)). Feedback was strongly positive in terms of discussion and understanding of treatment options, however demonstrated a more variable response as to personal priorities and comfort with decision making. A score of >37.5 is considered significant in terms of decisional uncertainty at the point of making. The median decisional regret score was 0, ranging from 0-22.

A median score of 6.6/10 (3-10) was given by surgeons in response to whether the reconstituted images could be useful in pre-operative surgical planning, and 7.1 (4-10) as to whether they were felt to be accurate. There was more positive feedback (8.6/10 (6-10)) with respect to a role in trainee education, and a desire to incorporate routinely into patient consultations (8.5/10 (6-10)). None of the surgeons changed their intra-operative plan after review of the 3D model. A thematic analysis of free text answers revealed: concerns over anatomical accuracy (due to the use of CT rather than MRI), failure to convey more nuanced-decision making (for example the necessity to resect structures for reasons other than direct tumour involvement), and financial implications. The use of the models to augment trainee education and patient discussion was, however, overwhelmingly encouraged.

Surgical fellows gave a median score of 9.0/10 (7-10) with respect to the use of the models as a training tool, with free-text comments highlighting the ability to talk through surgical steps with a visual aid and envisage areas for potential threat to clear margins. On review by a radiologist a mean score of 5.4/10 (2-10) was given in response to whether the reconstituted images were accurately segmented from the original CT imaging. It was reported that the position and size was accurate for exophytic lesions and nodal disease, however less accurate for lesions within the colonic lumen. One scan scored as only 2/10 included primary tumour and nodal metastasis within the same outline, rather than separate lesions. It was not felt that the models would be useful during the multi-disciplinary team cancer meeting, nor for training of radiology trainees.

**Figure 1:**
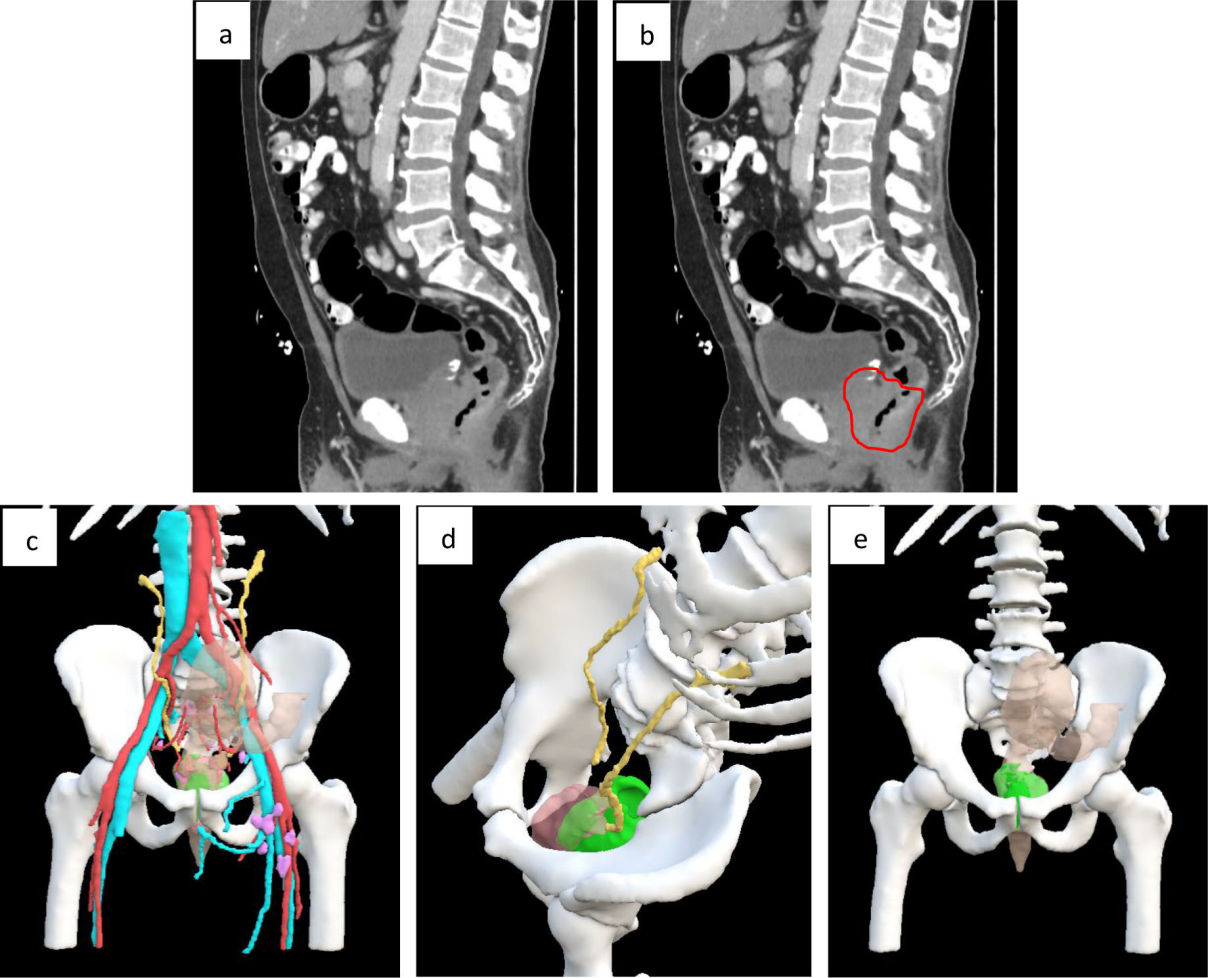
An example of the reconstituted images produced using the software. **(a)** A sagittal image of a CT scan demonstrating a locally advanced mucinous rectal cancer invading the prostate **(b)** A sagittal image of a CT scan demonstrating a locally advanced mucinous rectal cancer invading the prostate with tumour highlighted **(c)** An antero-posterior view of the reconstituted 3D image **(d)** An oblique view of the reconstituted 3D image **(e)** An antero-posterior view of the reconstituted image with additional anatomical structures removed to demonstrate the tumour along.

An example of the reconstituted images displayed on the software is demonstrated below in **Figure 1**.

## Discussion

This pilot study has demonstrated an invaluable benefit to patient education with the use of 3D visual aids during the consent process for surgery for advanced pelvic malignancy. Patient feedback was overwhelmingly positive, providing a greater level of understanding of the operation and making patients feeling empowered about their decisions. Shared decision making surrounding the management of advanced pelvic malignancy requires patients and surgeons to have complex conversations about surgical morbidity and long-term quality of life. The use of visual aids can help to facilitate those discussions and in periods of intense stress, serve as a memory aid (4, 5). The decisional conflict scale demonstrated an overall positive experience whilst using the models however was variable in its scoring. This again highlights the importance of utilising initiatives such as visual modelling to support challenging conversations.

A systematic review of studies utilising 3D modelling for rectal cancer found that both virtual reconstituted images and printed physical models were effective for surgical planning and the identification of areas at risk, particularly for recurrent or advanced rectal cancer or more challenging areas such as the pelvic side wall (6). Although the feedback from surgeons was cautious with regards to the use of this CT generated software for detailed operative planning due to the more detailed MRI imaging available, this study expands on this to report its potential as a training aid. Due to the low availability, ethical issues, and financial costs associated with cadaveric training, there has been a shift towards reproducible and widely available computer-based training. Pellino et al. demonstrated that for a rare retrorectal tumour, 3D modelling can enhance traditional training when used in pre-operative planning before conducting that procedure with cadaveric simulation (9).

The use of 3D imaging and printing in surgical planning and training is an expanding field, and the use of such technology is predicted to become widespread in the coming years (10). 3D modelling which can provide an accurate representation of specific anatomy whilst also displaying the position of vital neurovascular structures is crucial. As of yet, the inclusion of structures such as the autonomic nerves supplying the genitourinary tract has not been routinely achievable, but this has been reported with the use of reconstituted MRI scans (11). These features may further facilitate discussions surrounding potential morbidity that may directly impact upon underreported quality of life. As the evidence base for the use of the models potential expands, the choice of original imaging and use of MRI scanning can also be invested in. The time and financial implications of the production of 3D models must also be taken into account in a continually strained healthcare system, however there is an ever-expanding pool of literature which supports this being an invaluable resource worthy of further development and implementation. The design of further studies using 3D models to include a control group or pre and post-model scores would give a better understanding of the magnitude of benefit and potential role of expansion.

## Data Availability

The raw data supporting the conclusions of this article will be made available by the authors, without undue reservation.
